# Bevacizumab is associated with cerebral microstructural alterations: a DTI study in high-grade glioma

**DOI:** 10.3389/fneur.2023.1191226

**Published:** 2023-05-25

**Authors:** Rebecca Kassubek, Dorothée Lulé, Albert C. Ludolph, Jan Kassubek, Hans-Peter Müller

**Affiliations:** ^1^Department of Neurology, University of Ulm, Ulm, Germany; ^2^German Center for Neurodegenerative Diseases (DZNE), Ulm, Germany

**Keywords:** glioblastoma, bevacizumab, magnetic resonance imaging, diffusion tensor imaging, microstructural damage

## Abstract

**Background:**

For recurrent high-grade glioma, especially glioblastoma, no standard of care treatment exists. Due to the prolongation of progression-free survival and a cortiocosteroid-sparing effect, bevacizumab is often used in this condition. Despite initial clinical responses, there is growing evidence that bevacizumab may potentiate microstructural alterations which may cause cognitive decline, mostly affecting learning and memory.

**Methods:**

To investigate bevacizumab-associated microstructural damage of defined regions of interest (ROIs) in the white matter, diffusion tensor imaging (DTI) was performed in 10 patients with a case history or third-party report for neurological dysfunction concerning cognitive performance. Serial DTI data before and under bevacizumab were collected and longitudinal changes of fractional anisotropy (FA), axial diffusivity (AD), and radial diffusivity (RD) were assessed in mesiotemporal (hippocampal), frontal, and occipital regions.

**Results:**

The longitudinal DTI data under bevacizumab compared to DTI prior to bevacizumab demonstrated a significant decrease in FA and increase in AD and RD both in mesiotemporal (hippocampal) regions and in frontal regions, whereas occipital regions showed no significant alterations in DTI metrics.

**Conclusion:**

The regionally impaired microstructure in mesiotemporal (hippocampal) regions and in frontal regions is in line with the fact that neurocognitive impairment in learning and memory is mostly related to hippocampal integrity and attentional control in frontal regions. Further studies could investigate the potential of DTI to assess bevacizumab-associated microstructural damages in vulnerable brain regions.

## 1. Introduction

Glioblastoma multiforme (GBM) is the most common type of high-grade primary brain tumor and accounts for approximately 14% of all CNS tumors; it has an annual incidence rate of 3 per 1,00,000 inhabitants (1). Despite multidisciplinary treatment approaches, including maximal safe surgical resection, radiation therapy, and chemotherapy with alkylating agents (2), the prognosis is still very limited with progression-free survival (PFS) of 7–8 months and median overall survival of 14–16 months (3). To date, there is no widely accepted standard care for patients with recurrent GBM, and treatment options in this condition are re-resection, re-irradiation, and re-exposure to alkylating agents (2). Another frequently used agent in recurrent high-grade glioma, especially GBM, is bevacizumab, a humanized monoclonal anti-vascular endothelial growth factor (VEGF) antibody (4). VEGF expression is highly increased in high-grade glioma, leading to increased microvascular proliferation and thus facilitating tumor growth and invasion (5). Bevacizumab failed to show significant prolongation of overall survival in placebo-controlled trials, preventing approval of bevacizumab in the European Union for the treatment of newly diagnosed or recurrent GBM. Nevertheless, there is a widely accepted benefit of PFS (6–8). Several studies demonstrated that a subgroup may show long-term responses to bevacizumab of more than 1 year (9–11) with a corticosteroid-sparing effect (4).

Although generally well tolerated, there is some first evidence that bevacizumab might potentiate cognitive decline. Previous studies indicated that there is a clinical deterioration of cognitive capabilities in patients treated with bevacizumab (7, 12, 13). This decline may be associated with the observed significant decrease in whole brain volume and gray matter volume under current bevacizumab (14) and a progressive decrease in absolute hippocampal volume following bevacizumab treatment (15). One hypothesis for this observation might be that VEGF is not only supposed to stimulate angiogenesis but also acts as a trophic factor for neural stem cells and promotes neurogenesis (16). Neurogenesis plays an important role in structural plasticity and network maintenance which is essential for intact cognitive function, including learning and memory. Accordingly, altered neurogenesis (e.g., in the adult hippocampus) represents an early critical event in the course of severe cognitive decline in the sense of dementia (17). In the course of healthy cognitive aging, structural alterations and volume loss in the hippocampus is closely related to memory decline, whereas in frontal areas, it is associated with executive functions, including attentional control (18, 19).

The assessment of damage in neurogenesis under bevacizumab with routine neuroimaging tools is limited. Diffusion tensor imaging (DTI) with tractography is an advanced diffusion magnetic resonance imaging technique that allows for the analysis of the structural connectivity of the brain and can be used as a non-invasive marker to analyze white matter integrity, identifying white matter microstructural changes (20). Fractional anisotropy (FA), axial diffusivity (AD), and radial diffusivity (RD) are DTI metrics to analyze white matter microstructure (21). FA is sensitive to microstructural changes but does not indicate a specific type of lesion, while AD tends to be strongly affected by axonal injury, and RD is sensitive to white matter damage due to demyelination and less to changes in the axonal density or size (22–24).

Currently, there is a lack of knowledge regarding such microstructural damage in vulnerable brain regions following bevacizumab therapy. Nevertheless, any possible microstructural changes of functional relevance are of utmost importance to the patients and thus need to be further determined. The aim of our study was to apply DTI measurements (FA, AD, and RD) to analyze a possible effect of bevacizumab on regional white matter integrity in patients with recurrent high-grade glioma and to assess if bevacizumab treatment might be associated with microstructural alterations of distinct brain regions, including mesiotemporal (hippocampal) and frontal areas.

## 2. Methods

### 2.1. Patients

This case series study was approved by the Ethical Committee of the University of Ulm. All patients gave written informed consent for MRI acquisition in accordance with the Declaration of Helsinki. None of the patients had any contraindications against MRI scanning.

In total, 21 patients with high-grade glioma (WHO grades III and IV) were considered for this retrospective longitudinal study (180 MRI scanning sessions). All these patients were treated with bevacizumab as monotherapy, i.e., they received 10 mg per kg bodyweight intravenously every 14 days. From these 21 patients, those who had at least two complete MRI scanning visits (containing T2-weighted, fluid-attenuated inversion recovery (FLAIR), and DTI scans) without bevacizumab and a least two complete MRI scans under bevacizumab medication were selected for analysis. All patients subjectively complained of cognitive impairment, confirmed by their relatives or proxy in all cases, but since it was not part of the study protocol, no detailed neuropsycholocal testing was performed. Finally, longitudinal MRI data of 10 patients were analyzed; the details are given in the results and in Table 1.

**Table 1:** MRI scan statistics and patient characterization.

Patient	Age/years	Gender	MRI scans before bevacizumab medication	MRI scans under bevacizumab medication	Total observation period / months	Tumor localization
#1	70	M	2	2	8	Left parietal/occipital lobe
#2	53	M	4	6	31	Left frontal lobe
#3	59	F	4	3	16	Right frontal/temporal lobe
#4	69	M	2	3	10	Left temporal lobe
#5	55	M	7	6	35	Left frontal lobe
#6	75	M	2	3	9	Right temporal lobe
#7	42	F	6	2	14	Bi-frontal lobes
#8	49	F	6	6	24	Bi-frontal lobes
#9	69	M	2	3	12	Left temporal lobe/right parietal/occipital lobe
#10	70	M	3	5	16	Left parietal lobe
–	**61 ± 11**	**7 M/3F**	**4 ± 2**	**4 ± 2**	**18 ± 9**	

### 2.2. MRI protocol

MRI scanning was performed on a 1.5 Tesla Magnetom Symphony (Siemens Medical, Erlangen, Germany); the study protocol consisted of the following scans:

- contrast-enhanced T1w scan (magnetization-prepared rapid gradient-echo, MPRAGE) with 144 sagittal slices, 256 × 256 pixels, slice thickness 1.2 mm, pixel size 1.0 mm × 1.0 mm; the echo time (TE) and repetition time (TR) were 4.2 ms and 1,640 ms, respectively.
- diffusion tensor imaging (DTI) scan consisting of 52 volumes (64 slices, 128 × 128 pixels, slice thickness 2.8 mm, pixel size 2.0 mm × 2.0 mm) representing 48 gradient directions (b = 800 s/mm^2^) and four scans with gradient 0 (b = 0). TE and TR were 95 ms and 8,000 ms.
- FLAIR T2-weighted scan with 49 coronar slices, 512 × 448 pixels, slice thickness 3.0 mm, pixel size 0.43 mm × 0.43 mm; TE and TR were 82 ms and 8,500 ms.

### 2.3. Data analysis

The MRI analysis software *Tensor Imaging and Fiber Tracking* (TIFT) (25) was used for data processing. A standardized analysis cascade was applied, as previously described (26–28). After quality control for artifacts (29) and DTI data correction for eddy current distortions (30), all individual MRI data (3D-T1w, DTI, FLAIR) were transformed to an iso-voxel grid of 1 mm^3^ (which is essential for an optimized intra-subject alignment of longitudinal data) and then individually aligned to the AC-PC line by a rigid-brain transformation; optimum alignment was then performed by a rigid-brain conjugate simplex fitting procedure using half-way alignment (31). The transformation to a 1 × 1 × 1 mm^3^ iso-grid affects the absolute FA, AD, and RD values. Nevertheless, in order to obtain an optimized intra-subject alignment of longitudinal data, it is essential to transform the data into a high-resolution grid. However, this analysis step has been successfully performed in many studies, and, as all data were transformed in the same way, statistical comparisons become feasible (26, 27). FA, AD, and RD maps were calculated as DTI metrics to analyze white matter microstructure (21, 32, 33).

**Figure 1:**
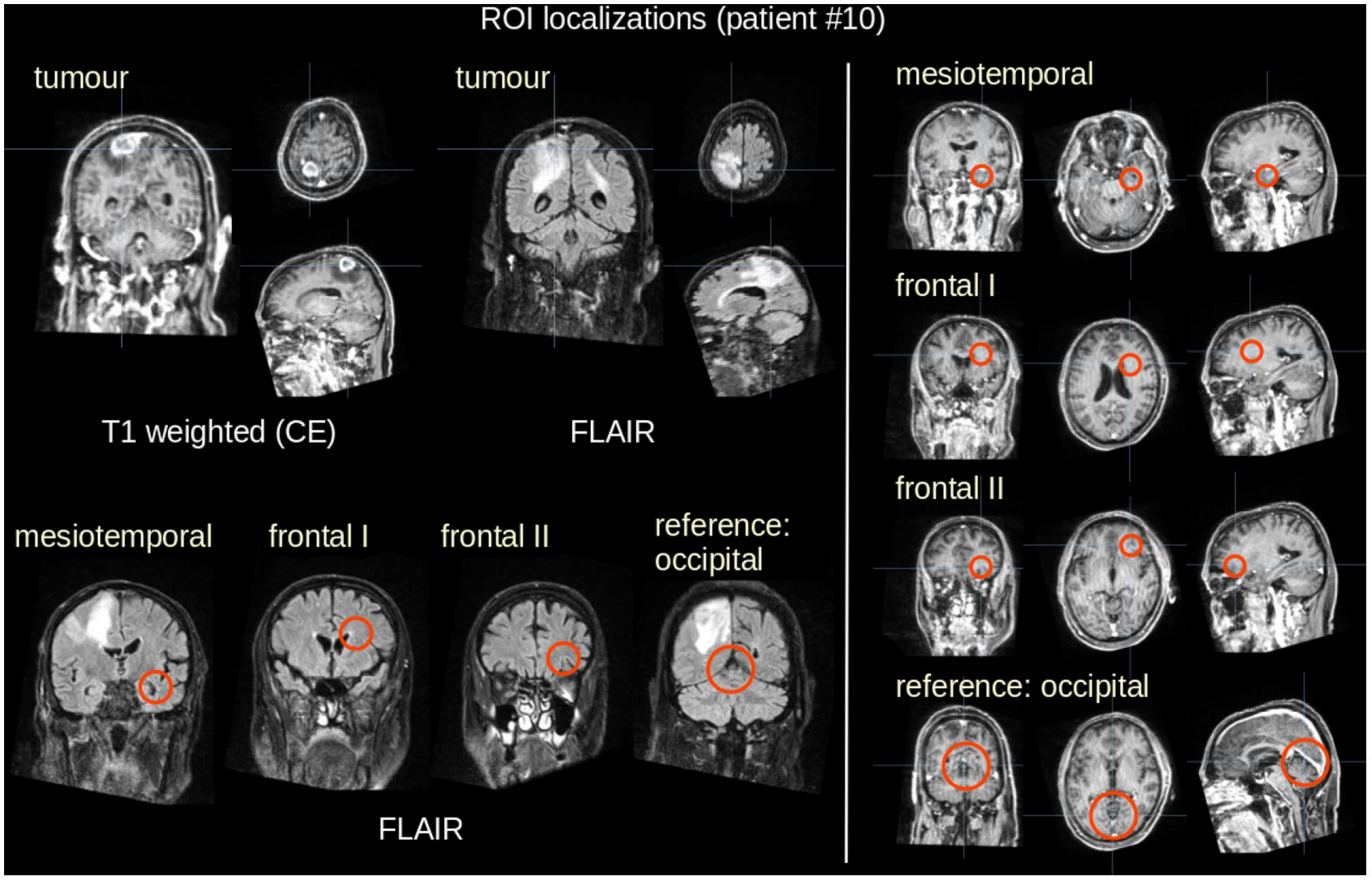
Example of 3D visualization (patient #10, 70  years, male). **Left panel**: Tumor localization in T1w (contrast-enhanced – CE) and in FLAIR data in coronar, axial, and sagittal representation (**upper panel**), and mesiotemporal (hippocampal) ROI localization (size 30 mm, **lower panel**). **Right panel**: ROI localizations in frontal areas (size 30 mm) and occipital (size 50 mm).

In the next step, ROI analysis was performed by setting spherical ROIs at predefined positions in the individually rigid-brain-aligned maps of DTI metrics. The atlas-based ROI positioning (34) was performed by two experienced neuroscientists (RK and HPM), i.e., in mesiotemporal (hippocampal) regions (ROI diameter 30 mm), in frontal regions (ROI diameter 30 mm), and as a reference region in post-occipital regions (ROI diameter 50 mm) (Figure 1). The medial temporal lobe (MTL) was chosen as it includes the hippocampus and the parahippocampal regions, which are crucial for episodic and spatial memory (35). The prefrontal cortex was included as it orchestrates higher cognitive functions and behavior in the sense of cognitive control (36). Post-occipital regions are primarily involved in visual processing and are thus subordinately involved in cognitive processes (37). ROI positioning in T1-weighted anatomical maps and in maps of DTI metrics (38) followed general procedures for ROI-based analyses (39), also described in detail by our group [e.g., (40)] where ROI positions have been analytically optimized in size and in position.

Average FA, AD, and RD values were calculated in these ROIs using an FA threshold of 0.2 [gray matter, CSF, and the tumor tissue shows FA values <0.2 (41)]; the average FA, AD, and RD values were then arithmetically averaged bihemispherically. Individual ROI localizations were assured in T1w scans and performed in FA, AD, and RD maps. Additionally, only ROIs were included in the analysis where no tumor involvement of the brain region could be detected in FLAIR data.

Differences in DTI metrics (∆FA, ∆AD, and ∆RD) were calculated for each subject by arithmetically averaging the ROI values from longitudinal data before bevacizumab medication and under bevacizumab medication, respectively. The bihemispherically averaged data were then analyzed by calculating the difference of averaged values before bevacizumab medication and under bevacizumab medication for each ROI localization, i.e., mesiotemporal (hippocampal) ROI, frontal ROIs, and in addition occipital ROI as a reference region. In order to provide the comparability between ROI-based values of FA maps in different subjects, statistical significance was tested by *t*-test after normalization of the individual absolute FA, AD, and RD values to the individual average value before bevacizumab medication had been performed.

### 2.4. Statistics

Statistical analysis was performed using standardized non-parametric testing. Data were presented as mean (arithmetic average) ± standard deviation. For the normally distributed samples (test for normal distribution was performed by the Kolmogorov–Smirnov test), comparisons between two groups (data “before” and “under bevacizumab medication”) were conducted using a two-tailed Student’s *t*-test. A two-tailed value of p of less than 0.05 was considered statistically significant.

## 3. Results

After the application of the inclusion and exclusion criteria, longitudinal MRI data of 10 patients (7 male/3 female) were included in the analysis; for the statistics of the included subjects and scans refer to Table 1. Of those 10 patients, seven had been diagnosed with glioblastoma, IDH wildtype, or not otherwise specified (NOS) and three with anaplastic astrocytoma, NOS, according to either the third or the fourth edition of the WHO classification of CNS tumors. Visual inspection of FLAIR images revealed that there was no general increase in white matter hyperintensities after the start of bevacizumab therapy.

**Figure 2:**
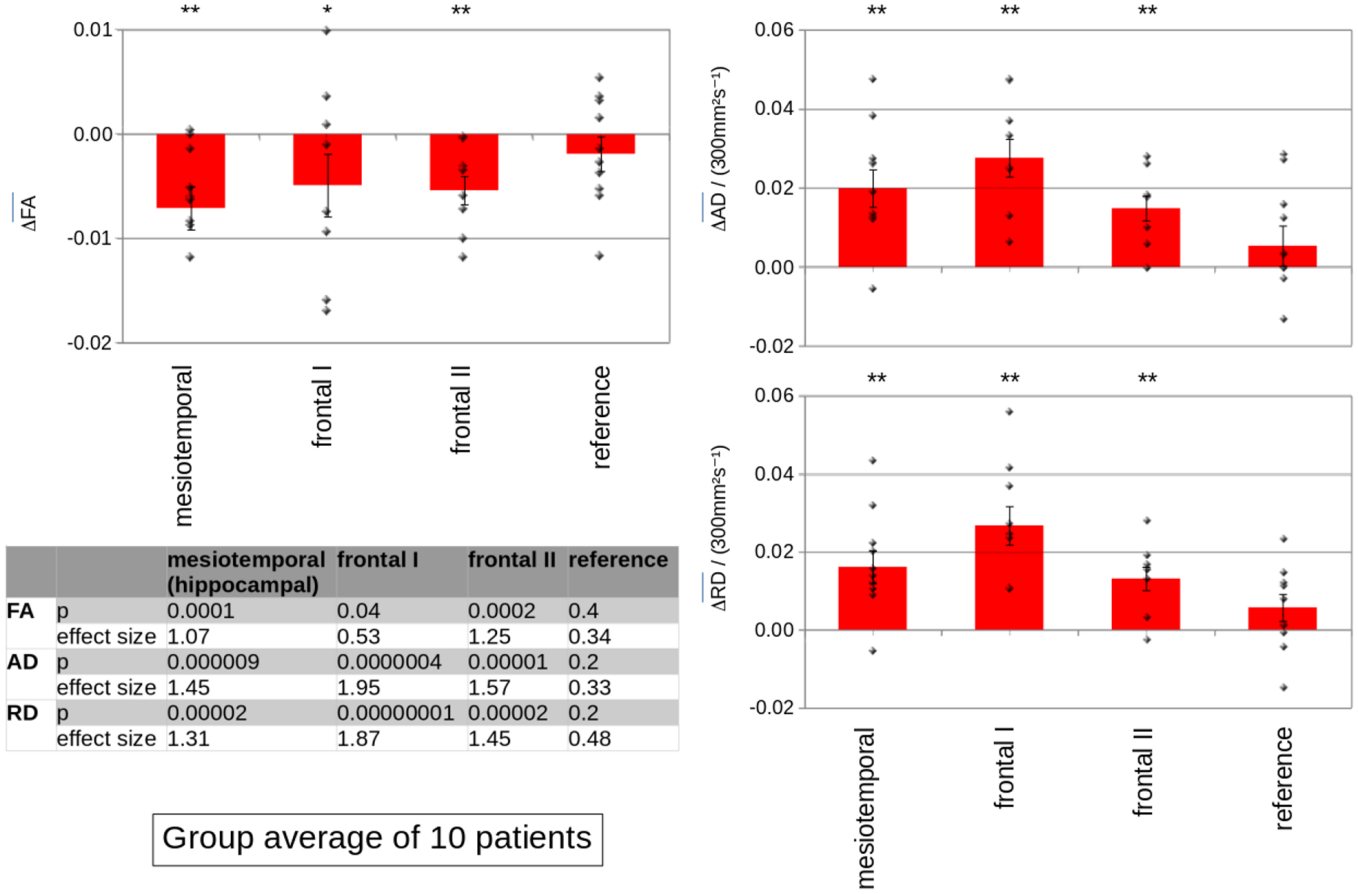
Group averaged differences of the DTI metrics FA (**left panel**), AD (**right upper panel**), and RD (**right lower panel**) between the MRI prior to bevacizumab medication and the MRI under bevacizumab therapy for the analysis of mesiotemporal (hippocampal) ROIs, ROIs in the two frontal regions, and in the reference region. Significant differences in DTI metrics (∆FA, ∆AD, and ∆RD) were observed in mesiotemporal (hippocampal) ROIs as well as in frontal ROIs, whereas differences were not significant in the occipital reference region. Error bars are the standard error of the mean (SEM); significance was tested for intra-subject normalized mean values, ** *p* < 0.001; * *p* < 0.01.

At the group level, the group averaged FA decrease was 2.7% in the mesiotemporal (hippocampal) region, 1.8% in frontal ROI I, and 2.1% in frontal ROI II, whereas the FA decrease in the occipital region was 0.7% (Figure 2, left panel). Group averaged AD increase was 4.5% in the mesiotemporal (hippocampal) region, 6.8% in frontal ROI I, and 3.8% in frontal ROI II, whereas the AD increase in the occipital region was 1.4% (Figure 2, right upper panel); group averaged RD increase was 4.7% in the mesiotemporal (hippocampal) region, 9.1% in frontal ROI I, and 4.5% in frontal ROI II, whereas the RD increase in the occipital region was 1.8% (Figure 2, right lower panel). In order to add further information about the hippocampus as a structure of specific interest, the hippocampus was separately investigated with an ROI of 20 mm in addition to the mesiotemporal region. The results were similar, i.e., regional FA decrease and AD and RD increase, respectively, were observed in a similar magnitude (Supplementary Figure 1).

Summarizing these results, significant differences between the data before and under bevacizumab medication were observed in (inter-subject) normalized averaged FA, AD, and RD values at the group level in the mesiotemporal (hippocampal) region as well as in frontal ROIs, whereas FA, AD, and RD did not differ significantly in the occipital reference region. NB: Only ROIs contributed to the analysis where no tumor involvement of the brain region could be detected in FLAIR data—that way, frontal areas I and II of patients #7 and #8 did not contribute to the analysis; in summary, out of the 80 analyzed ROIs (10 patients × 4 ROIs × 2 hemispheres), 17 ROIs had to be excluded due to tumor involvement.

**Figure 3:**
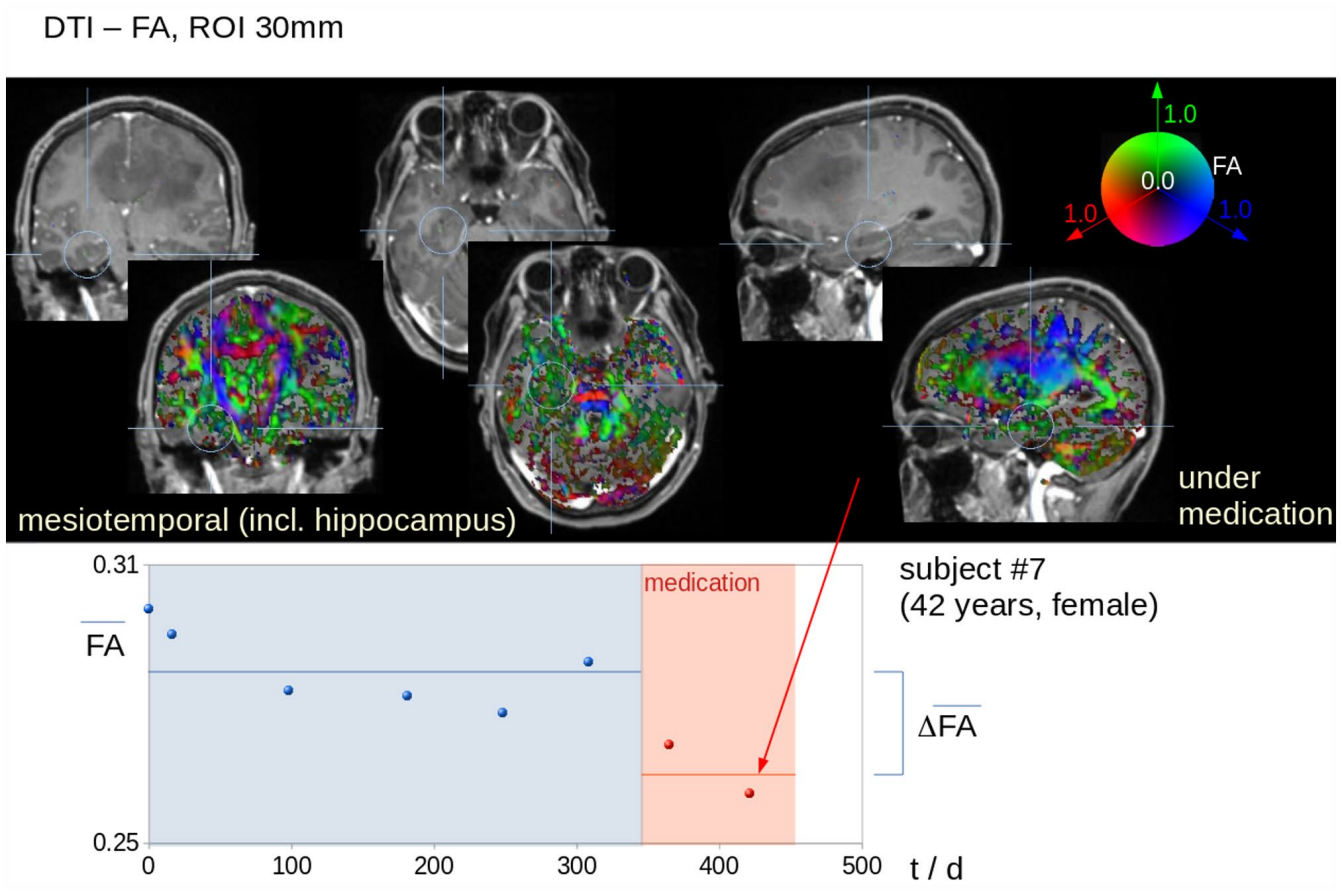
Individual example (patient #7, 42  years, female) for mesiotemporal (hippocampal) FA ROI analysis. **Upper panel**: ROI localization in the FA maps. The position of the mesiotemporal ROIs include the hippocampus as visualized in the T1-weighted images (contrast agent) and in the color-coded FA maps, that way focussing on brain areas in which a change reflecting a cognitive decline is hypothesized. The threshold of 0.2 (as applied in the color-coded FA maps) shows that gray matter as well as CSF was excluded from the analyses, that way focussing only on white matter alterations. **Lower panel**: Average FA values in the left mesiotemporal (hippocampal) ROI show a decrease under bevacizumab therapy.

**Figure 4:**
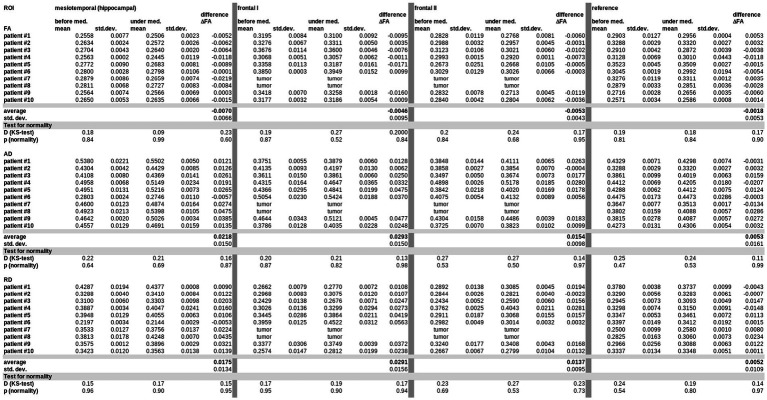
Differences in DTI metrics (mean FA, mean AD, mean RD) with standard deviations (std. dev.) in all ROIs for all subjects (before and under bevacizumap medication). Means and differences were tested for normal distribution (Kolmogorov–Smirnov (K–S) test).

Figure 3 shows an individual example of MRI data analysis with differences in FA maps in the left mesiotemporal (hippocampal) ROI (where no tumor involvement of the brain region could be detected in FLAIR data) before and under bevacizumab medication. Up to seven MRI scans before the start of bevacizumab were included; the inter-subject distribution in each ROI, separately before and under bevacizumab medication, was tested for normal distribution (Figure 4). In these data, DTI metrics showed a standard deviation that was lower or up to the same range as the differences of averaged values before and under bevacizumab medication.

Differences in DTI metrics were averaged for maps before bevacizumab medication on the one hand and maps under bevacizumab on the other hand, independent of the number of available data (visits/scans) before or under bevacizumab medication, respectively. That way, individual DTI metrics alterations (∆FA, ∆AD, and ∆RD) could be obtained for each patient separately (example in Figure 3). To control for the potential confounder that our data might contain substantial changes due to previous therapies, we have included only subjects with at least two (and up to seven) data sets before bevacizumab treatment to use these as a baseline since they included potential effects of previous therapeutic interventions, especially irradiation. Significant longitudinal alterations in DTI metrics in this data set prior to bevacizumab treatment could not be observed. For this reason, it seems safe to assume that the significant alterations in DTI metrics after the start of bevacizumab are not associated with the prior therapies. A detailed summary of all therapies prior to bevacizumab for each patient is listed in Supplementary Table 1.

Additionally, FA alterations were investigated with respect to associations (correlation analyses) with age as a contributing factor to cognition. However, no significant association between DTI metrics (FA, AD, and RD) and age could be detected.

## 4. Discussion

Although bevacizumab failed to show prolongation of overall survival of patients with GBM in large studies (42), there is a broad acceptance of bevacizumab in recurrent high-grade glioma because bevacizumab seems to have beneficial effects on progression-free as well as deterioration-free survival and steroid-sparing effects [(42–44)] and on quality of life in recurrent tumor (45). Taken together, prolonging PFS and therefore ideally prolonging the time to the loss of personal and economic independence is a worthwhile ambition for the therapeutic team. The systemic toxicity profile is well described and mostly manageable with proteinuria, bleeding, and thrombembolic events, and also hypertension as most frequent adverse events (46).

Despite those encouraging effects on PFS, there is also some evidence that after initial benefits on clinical parameters, bevacizumab may lead to a decline in neurocognitive function and quality of life despite the absence of radiographical or clinical signs of tumor progression (7, 47). As an imaging correlate of this decline of neurocognitive function associated with bevacizumab treatment, there has been evidence of structural brain alterations, especially in the hippocampal volume (14, 48) with a probable effect of VEGF on neurogenesis (16).

The technical approach of DTI was used in the current study since this application to diffusion-weighted MRI enables the assessment of microstructural properties of white matter fiber systems by measuring the differences in constraints on water diffusion in different types of tissue (49) so that DTI and tractography allow for the *in vivo* reconstruction of the brain’s white matter connections for the mapping of the structural connectivity using measures of connectivity or tissue microstructure (50). DTI has been successfully applied to the analysis of processes like brain aging (32) and to a multitude of brain pathologies including a study on patients with high-grade glioma to detect side-effects on the patients’ cognitive function (39). By ROI analysis, Rydelius et al. reported reduced FA in the corpus callosum, the centrum semiovale, the hippocampus, and the amygdala (39). Based on these analysis methods, the current study investigated in a hypothesis-guided approach bevacizumab-associated alterations of DTI metrics in ROIs located in mesiotemporal (hippocampal) and frontal areas compared to ROIs in occipital areas as the reference. The comparison of the longitudinal DTI data under bevacizumab with DTI prior to bevacizumab demonstrated significant changes for FA and AD and RD both in the mesiotemporal (hippocampal) and in the frontal regions, whereas the occipital reference ROI showed no significant differences for all metrics. In detail, the decrease in FA was slightly more pronounced in the mesiotemporal (hippocampal) ROI, while the differences for AD and RD were most prominent in the frontal regions. These subtle differences in the regional pattern between the DTI metrics might reflect their differences in analysis target, but it can be held for all DTI metrics that both neurocognitively relevant regions (mesiotemporally and frontally) according to the hypothesis were found to show a change in microstructure after the start of bevacizumab. It seems premature at this point to classify the observed changes in microstructure with respect to axonal lesions or demyelination.

This regional pattern is in line with the fact that neurocognitive impairment in learning and memory is mostly related to hippocampal integrity and frontal regions to attentional control (18, 19) known to be negatively affected by bevacizumab treatment (7). In contrast to the loss of hippocampal volume that could be observed after 6 months and was progressive over years under continuous bevacizumab treatment (15), an alteration of DTI metrics could already be observed within the first MRI scan after the initialization of bevacizumab. Therefore, DTI might be used as an early biomarker for bevacizumab therapy-associated microstructural changes. These changes could explain some of the cognitive impairments in the context of bevacizumab treatment reported in the literature.

The findings should be considered in the context of several limitations mostly due to the limited sample size with subjects who are heterogeneous with regard to tumor site. However, the high number of 77 MRI scans from the longitudinal acquisitions that contributed to the results statistically compensated for this number. Initially, a total of 180 MRI scans from 21 patients were included in the analysis. The final analysis was limited to 10 patients according to strict inclusion criteria, resulting in 77 scans that contributed to the results. The selection process included that at least two scans in both conditions (before or under medication) were available; as a final quality control in each subject, averaged DTI metrics before medication and under medication, respectively, showed longitudinal differences that were in the same range as the standard deviation before medication and under medication, respectively. Moreover, positive results within limited sample sizes may be considered to be even more robust under certain circumstances (51) which underlines the importance of the findings in the hereby described population. Therefore, the strict pre-selection of contributing data leads to the possibility of statistically comparing means. Nevertheless, longitudinal measures of within-subject effects provide highly valuable information on a true bevacizumab effect which minimizes confounders of intrasubject variance regarding heterogeneity in clinics.

A further limitation was the lack of a control cohort without medication. However, due to the retrospective character of this study, a control cohort without any medication was not available. Instead, we analyzed ROIs longitudinally in each subject separately and calculated differences before and under medication in different brain regions. Therefore, in the analyzed patient group, DTI metrics alterations were identified both in mesiotemporal (hippocampal) and frontal areas, whereas no alterations were reported in occipital regions which served as a reference. Thus, the reference data were the intra-subject patient comparisons. To minimize direct tumor-associated effects, we did not include regions with any signs of tumor in T2/FLAIR-weighted or post-contrast T1 weighted sequences. Due to the longitudinal concept and the appropriate selection of data, at least two scans before and two scans under bevacizumab medication were investigated. Therefore, the read-outs were the DTI metrics alterations over time in the longitudinal data, and thus, additional controls or reference data are not needed to contribute to the results. One major limitation is that due to the retrospective character of this study, no dedicated formal neuropsychological assessments were performed in the context of this study for a correlation analysis although it has to be noted that for every case there was a case history or third-party report for neurological dysfunction concerning cognitive performance. Accordingly, all patients subjectively complained of cognitive impairment, confirmed by their relatives or proxy. Furthermore, an effect of treatments prior to bevacizumab cannot be excluded as all patients were pretreated with irradiation and chemotherapy with alkylating agents. Nevertheless, we included up to seven MRI scans before the start of bevacizumab in which no significant changes in DTI metrics could be observed despite these treatments. However, an effect on brain volume was seen immediately after the start of bevacizumab; thus, the effect of the prior treatments might be considered to be of limited if any effect on the results. Finally, all data were acquired on a 1.5 T system which has a lower signal-to-noise ratio compared to a field strength of 3.0 T.

In summary, DTI may serve as a non-invasive biological marker to map bevacizumab-induced microstructural damage in vulnerable brain regions, i.e., mesiotemporal (hippocampal) and frontal areas, in patients with recurrent high-grade glioma. These alterations of structural integrity might be regarded as correlates to the clinical observation of memory decline and loss of executive functions (attentional control) under therapy with the VEGF antibody due to inhibition of VEGF’s role as a promotor of neurogenesis and of neural plasticity. A prospective trial in which DTI analyses together with neurocognitive screenings is needed to confirm the functional effect on cognitive performance of the structural alterations and to further evaluate the role of DTI to determine microstructural alterations under therapy with bevacizumab. Therefore, future prospective studies with defined medication and detailed cognitive assessments should provide reliable information on bevacizumab-induced damage in vulnerable brain regions.

## Data availability statement

The data presented in this study are available from the corresponding author on reasonable request.

## Ethics statement

The studies involving human participants were reviewed and approved by the Ethical Committee of the University of Ulm. The patients/participants provided their written informed consent to participate in this study.

## Author contributions

RK, JK, and H-PM: conceptualization and writing—original draft preparation. RK and H-PM: investigation. H-PM: data curation. RK, DL, AL, JK, and H-PM: writing—review and editing. RK: project administration. All authors contributed to the article and approved the submitted version.

## Conflict of interest

The authors declare that the research was conducted in the absence of any commercial or financial relationships that could be construed as a potential conflict of interest.

## Publisher’s note

All claims expressed in this article are solely those of the authors and do not necessarily represent those of their affiliated organizations, or those of the publisher, the editors and the reviewers. Any product that may be evaluated in this article, or claim that may be made by its manufacturer, is not guaranteed or endorsed by the publisher.
