# Supplementary material for: Bevacizumab is associated with cerebral microstructural alterations: a DTI study in high-grade glioma
Source: Front Neurol. 2023 May 25;14:1191226. doi: 10.3389/fneur.2023.1191226 (PMC10247958; doi:10.3389/fneur.2023.1191226)
Supplement: Supplementary file 1 [file Data_Sheet_1.pdf]

patient	radiation therapy	chemotherapeutic agents used	Tumor Treating Fields
#1	60Gy*; 45Gy	temozolomide; lomustine	yes
#2	60Gy*	temozolomide	yes
#3	60Gy*; 39.6Gy	temozolomide; lomustine	no
#4	60Gy*; 45Gy	temozolomide; lomustine	no
#5	60Gy; 39.6Gy	temozolomide; lomustine/procarbazine/vincristine	no
#6	60Gy*; 45Gy	temozolomide; lomustine/etoposide	yes
#7	45Gy*	temozolomide	no
#8	60Gy*; 39.6Gy	temozolomide; lomustine	yes
#9	60Gy*; 36Gy	temozolomide	no
#10	60Gy	temozolomide	no

Supplementary Table 1: Therapeutic regimes prior to bevacizumab. “*“ denotes radiation therapy with concomitant and adjuvant chemotherapy with temozolomide; “;“ separates different therapeutic lines; “/” denotes combined chemotherapy
