# Supplementary figures and images for: Bevacizumab is associated with cerebral microstructural alterations: a DTI study in high-grade glioma
Source: Front Neurol. 2023 May 25;14:1191226. doi: 10.3389/fneur.2023.1191226 (PMC10247958; doi:10.3389/fneur.2023.1191226)

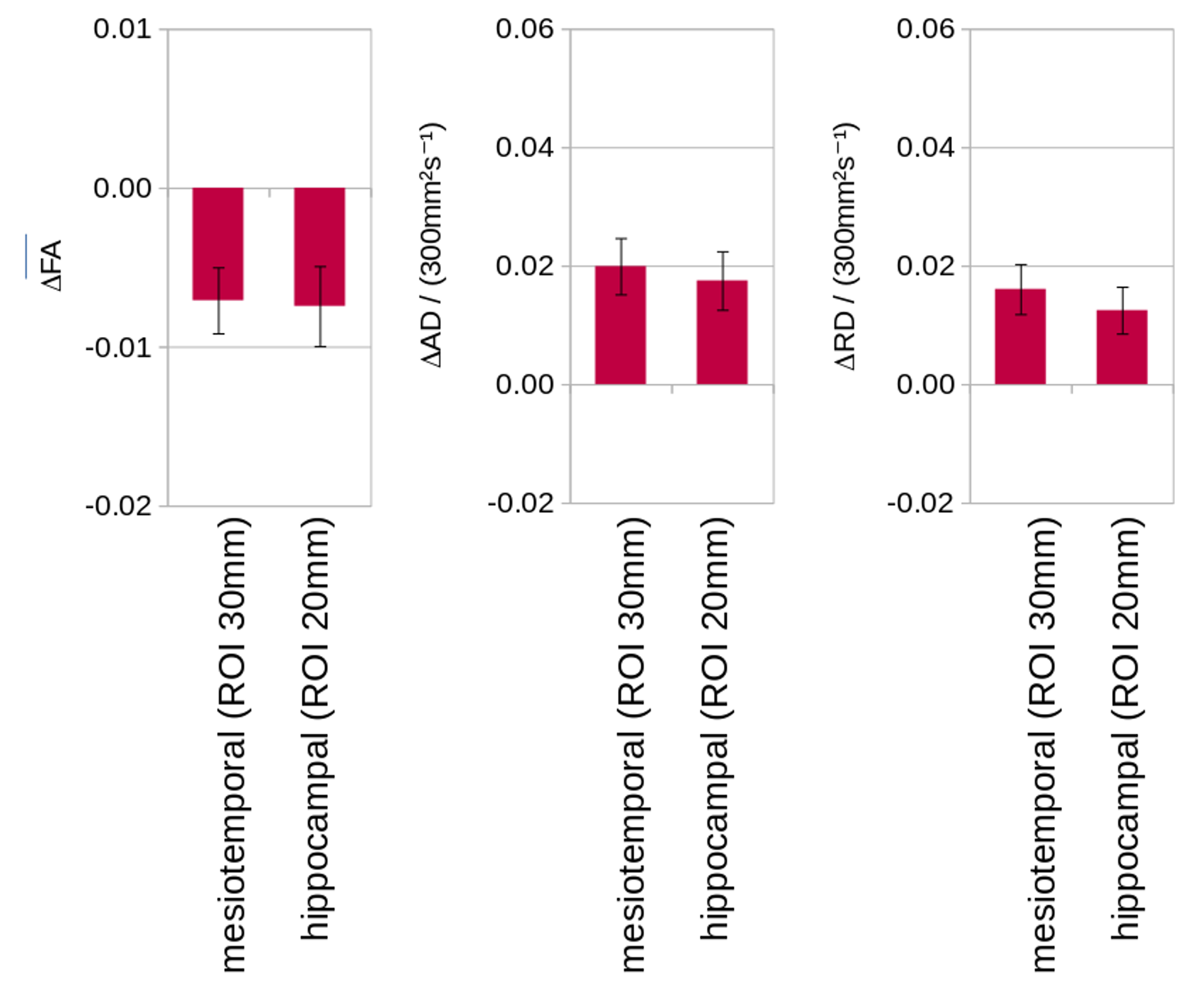

Supplement: Supplementary file 2 [file Image_1.TIF]
